# PaIR: Partition-Based Information Rebalancing for Robust Text-Based Person Search

**DOI:** 10.3390/jimaging12090400

**Published:** 2026-08-25

**Authors:** Luda Wang, Jiabao Li, Xinpan Yuan, Ningdan Zhang

**Affiliations:** 1 School of Computer Science and Science, Xiangnan University, Chenzhou 423000, China; luda_wang@163.com (L.W.);; 2 School of Computer Science and Science, Hunan University of Technology, Zhuzhou 412000, China

**Keywords:** text-based person search, information rebalancing, part alignment, semantic completion, cross-modal retrieval

## Abstract

Text-based person search (TPS) suffers from cross-modal informational skewness: pedestrian images are high-dimensional and redundancy-prone, while textual descriptions are sparse, incomplete, and sometimes inaccurate. To address the low alignment accuracy and poor robustness caused by the inherent uneven information distribution of visual and textual modalities in TPS, this paper proposes a unified Partition-based Information Rebalancing (PaIR) framework to realize balanced optimization and precise alignment of cross-modal information from both global content and local part dimensions. The framework adopts the CLIP dual-modal encoder for basic feature extraction and constructs a parallel global–local dual representation system to compensate for the lack of fine-grained spatial information in single global features. To eliminate modal redundancy and noise interference, a dual-modal noise suppression module is designed to filter invalid redundant information through visual foreground–background separation and textual token weight screening, while introducing adversarial constraints and orthogonal constraints to purify effective features. On this basis, a part balance alignment module is built to complete human semantic part decomposition and soft matching alignment for dual-modal features. Aiming at the common part semantic missing problem in textual descriptions, a visual part correlation affinity matrix is utilized for semantic associative completion to balance the information density of dual modalities. Finally, a global–local joint alignment strategy integrates hierarchical features and bidirectional cross-modal attention interaction to eliminate global–local semantic discontinuity and enhance fine-grained cross-modal matching capability. Extensive experiments on three public benchmarks demonstrate that PaIR consistently improves multiple baselines.

## 1. Introduction

Text-based person search (TPS) is a core research direction in the field of cross-modal retrieval. It aims to accurately retrieve target pedestrians from large-scale pedestrian image galleries through natural language descriptions, and is widely applied in real-world scenarios such as intelligent security monitoring, multimedia content retrieval, and human–computer interaction [1,2,3,4,5]. Different from traditional person re-identification tasks that rely solely on visual-only information, TPS breaks through the limitations of pure visual retrieval, builds a cross-modal semantic correlation bridge between visual images and natural language, and is more adaptable to flexible and complex manual retrieval requirements [6]. Current mainstream TPS methods are mainly optimized in three directions: global feature alignment, part-based modeling matching, and cross-modal attention interaction, which have effectively promoted the performance of cross-modal semantic matching. However, the alignment robustness defects caused by inherent modal differences have not been fundamentally resolved, restricting the practical application of TPS models in complex scenarios [7,8,9].

Existing studies generally overlook the core problem of cross-modal informational skewness in TPS tasks, which is a key bottleneck restricting the fine-grained retrieval capability of models [8,10]. In the visual modality, pedestrian images are high-dimensional feature representations, which not only contain effective features required for pedestrian identity discrimination, but also mix with a large amount of invalid noise information such as background redundancy, occlusion, and illumination interference, resulting in strong visual feature redundancy and scattered effective semantics [4,11]. In contrast, natural language descriptions of the text modality are inherently sparse. Manually annotated texts usually only cover local pedestrian attributes, with incomplete feature description, ambiguous semantics, and contradictory expressions, leading to missing semantic dimensions and insufficient information density of text features [3,12]. This structural imbalance of dual-modal information distribution directly causes cross-modal semantic mismatch: visual representations are dominated by redundant noise, while text representations lack complete semantic support for fine-grained alignment. Traditional global holistic alignment and uniform part segmentation strategies cannot effectively eliminate this skewness [2,13], making the model’s retrieval performance drop significantly when facing ambiguous and incomplete text descriptions, and failing to adapt to complex real-world scenarios.

**Figure 1:**
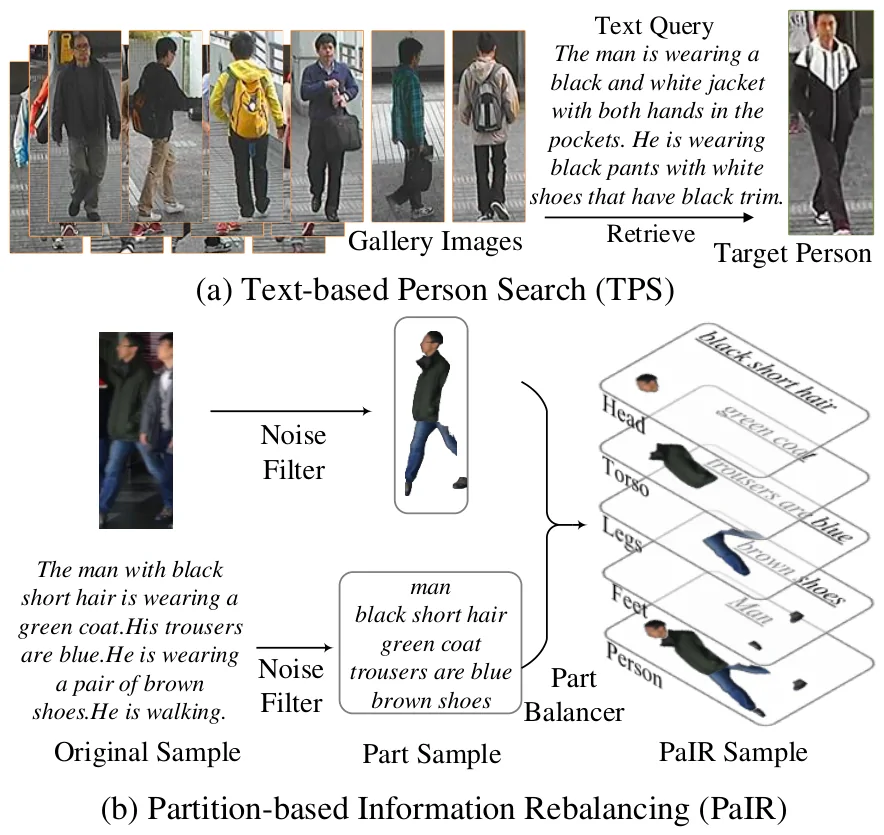
(**a**) Text-based person search (TPS) aims to locate the target person in gallery images from a textual query. (**b**) Our PaIR framework tackles cross-modal skewness by distilling redundant noise and enforcing part-level alignment, yielding cleaner semantics and more accurate retrieval.

**Figure 2:**
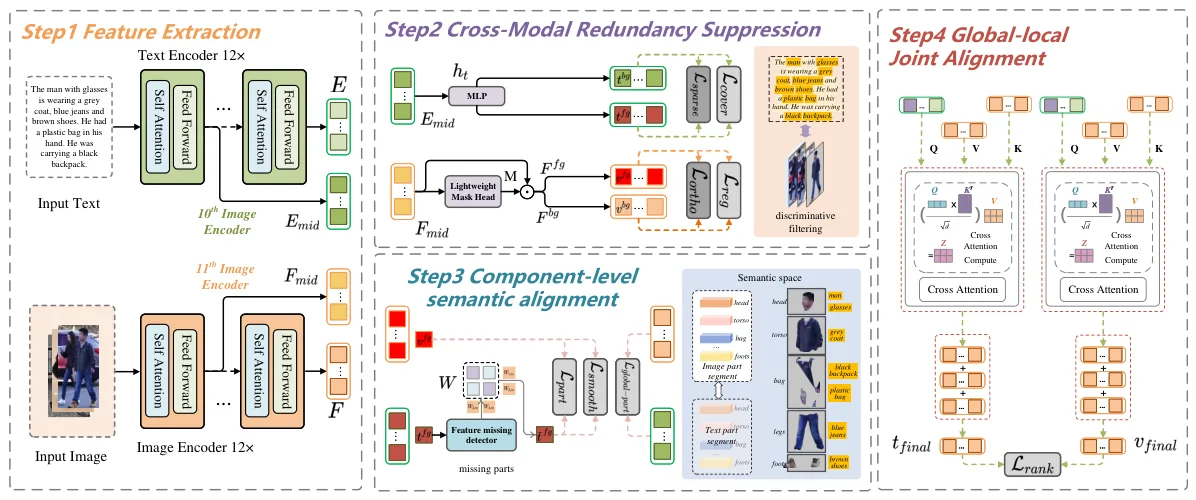
Overall framework of PaIR. The pipeline is organized into four stages: Feature Extraction, Cross-Modal Redundancy Suppression, component-level semantic alignment and global–local joint alignment.

To solve the core problems caused by cross-modal informational skewness, this paper proposes a Partition-based Information Rebalancing (PaIR) framework for dual-modal information balance optimization, as illustrated in Figure 1 and Figure 2, achieving systematic dual-modal information purification, balanced optimization and fine-grained alignment at both global semantic and local part levels. As shown in Figure 2, the framework first adopts the CLIP dual-modal pre-trained encoder to extract high-level global features and middle-layer Transformer features (the 11th layer for the visual encoder and the 10th layer for the textual encoder), thereby constructing a parallel global–local dual representation system that balances global semantic integrity and local fine-grained spatial details while overcoming the limitations of single-scale feature representation. Secondly, a dual-modal noise suppression module is designed to eliminate modal redundancy and noise interference. In the visual branch, a lightweight mask prediction head separates pedestrian foreground effective features from background redundant noise, and adversarial learning with gradient reversal layer and feature orthogonal constraints are adopted to purify identity-related visual features. In the text branch, a gated MLP performs token-level adaptive weight screening to filter invalid and ambiguous semantic noise, optimizing the effective information ratio of dual modalities at the source. Meanwhile, a part balance alignment module is constructed to decompose the purified dual-modal features into four human semantic parts (head, torso, legs and accessories) and realize precise part-level soft matching alignment. To address the ubiquitous problem of missing part semantics in textual annotations, a visual part semantic correlation affinity matrix is introduced for associative semantic completion, effectively compensating for the inherent sparsity and incompleteness of textual modality. Finally, the global–local joint alignment module fuses normalized global backbone features, local purified foreground features and bidirectional cross-modal attention interactive features to eliminate semantic discontinuity between global and local representations, realizing comprehensive and robust fine-grained cross-modal semantic alignment for TPS tasks.

Extensive comparative experiments, ablation studies and visual analysis are conducted on three mainstream public TPS datasets including CUHK-PEDES, ICFG-PEDES, and RSTPReid [1,4,14] to verify the rationality and effectiveness of each core module of PaIR. The experimental results show that the proposed framework delivers competitive performance across these benchmarks, with particularly strong results on the Top-5 and Top-10 metrics, while remaining close to the best-performing methods on Top-1. This observation supports the effectiveness and robustness of the proposed information rebalancing strategy. The core research contributions of this paper are summarized as follows: First, this paper systematically defines and analyzes the long-neglected cross-modal informational skewness problem in TPS tasks, revealing the essential negative impact of unbalanced modal information distribution on fine-grained semantic alignment and real-world retrieval robustness, which makes up for the cognitive deficiencies of existing research. Second, this paper innovatively proposes an integrated PaIR unified framework integrating dual-modal noise suppression, part-level information balance, visual-guided textual semantic completion, and global–local joint alignment, breaking the limitation of single-modality unilateral optimization and realizing systematic balanced optimization and precise matching of dual-modal information. Third, comprehensive experiments on multiple standard benchmarks verify the universality and superiority of the proposed method, providing an effective and feasible technical solution for solving cross-modal information imbalance and improving the robustness of fine-grained text-based person retrieval.

In summary, to tackle the issues of insufficient alignment accuracy and weak scene robustness caused by cross-modal informational skewness in text-based person search, this paper proposes the Partition-based Information Rebalancing (PaIR) framework for systematic optimization. The core contributions of this work are concluded as follows:

- We explicitly define and thoroughly analyze the long-neglected cross-modal informational skewness problem in TPS tasks, and reveal the restrictive mechanism of visual redundancy and textual sparsity on fine-grained matching.
- We design a complete technical path including dual-modal joint denoising, part-level balanced alignment, and visual-guided semantic completion. The proposed PaIR framework achieves competitive and often leading performance across three benchmark datasets, with strong generality and robustness.

## 2. Related Works

### 2.1. Text-Based Person Search

Text-based person search (TPS) aims to retrieve target pedestrian images from gallery sets according to open natural language descriptions, and has become a core problem in cross-modal retrieval and intelligent surveillance. Early TPS methods mainly relied on global embedding learning and attribute-guided matching, which improved coarse semantic alignment but struggled to capture subtle body-part cues and fine-grained phrase–region correspondence. Representative works such as Aggarwal et al. [15] and Ding et al. [1] explored attribute-aided reasoning and part-level modeling, showing that local structural information is essential for accurate retrieval.

In recent years, the rapid development of vision–language pre-training has significantly advanced TPS. Cao et al. [10] conducted an empirical study on CLIP-based person search and verified the strong transferability of large-scale pre-trained models. Shao et al. [2] introduced granularity-unified representation learning to reduce semantic mismatch across different levels of detail, while Yan et al. [6] proposed CFINE to strengthen fine-grained alignment under CLIP-based representations. More recent methods such as HiMo-CLIP [7] and FG-CLIP 2 [8] further enhance long-text reasoning and hierarchical semantic modeling. In parallel, Transformer-based and relation-aware approaches such as IVT [16], LCR2S [17], RaSa [18], and IRRA [19] improve cross-modal interaction from different perspectives, including global context modeling, region selection, relation reasoning, and CLIP-driven alignment. More recent works including CFAM [20], RDE [3], ICL [5], and DiCo [21] focus on robust fine-grained matching, noise-aware optimization, and more stable semantic fusion. Although these methods achieve strong performance, most of them still treat visual redundancy and textual sparsity in a relatively unilateral way, and therefore do not fully address the inherent cross-modal information imbalance that limits retrieval robustness in complex scenes.

### 2.2. Fine-Grained Alignment and Multimodal Structure Learning

Fine-grained cross-modal alignment is the core prerequisite for high-precision TPS retrieval, and multimodal structural semantic learning has become a mainstream technical route to break through the bottleneck of coarse matching performance in recent years. Traditional fine-grained methods mostly rely on fixed partitioning or attention weighting to mine local correspondences, but fail to model the structural syntactic relationship of text itself. Niu et al. [13] proposed textual dependency embedding for person search by language, which introduces a syntactic dependency structure to encode textual semantic relations and improves the accuracy of phrase–region alignment. Parcalabescu et al. [22] constructed a linguistic phenomenon benchmark for vision–language models and verified that the syntactic structure perception ability of multimodal models is positively correlated with fine-grained retrieval performance, emphasizing the importance of structural semantic learning for cross-modal tasks.

The latest studies from 2025 to 2026 further optimize structural fine-grained alignment from the perspectives of multi-scale feature fusion and semantic complementarity. Shao et al. [2] proposed granularity-unified representation learning, which constructs hierarchical cross-modal interaction at both global and local scales to alleviate semantic mismatch caused by single-scale feature alignment. From the perspective of data structure optimization, some studies adopt syntactic parsing and large language model generation to expand fine-grained textual data, alleviating the problem of insufficient detailed descriptions in original datasets [12]. However, most current structure learning methods only focus on strengthening feature alignment capability, without considering the unbalanced information density between visual and textual modalities. They lack effective suppression of visual redundant noise and targeted completion of missing textual semantics, which cannot fundamentally solve the structural mismatch problem caused by modal information skewness. Different from the above works, this paper innovatively combines noise filtering, part structural alignment and semantic associative completion to realize balanced optimization of multimodal structure and information density.

### 2.3. Multimodal Information Rebalancing and Noise Suppression

Modal information imbalance and noise interference are key obstacles restricting robust cross-modal retrieval, and research on information rebalancing and noise suppression has gradually attracted extensive attention in TPS and cross-modal retrieval fields. In terms of noisy correspondence learning, Hu et al. [23] studied the partially mismatched pair problem in cross-modal retrieval and derived a robust cross-modal learning framework with an unbiased risk estimator, which reduces the interference of mismatched samples on model training. Qin et al. [3] designed noisy correspondence learning for text-to-image person re-identification, which improves the model’s tolerance for inaccurate textual descriptions. In terms of visual noise suppression, Zhu et al. [4] proposed deep surroundings–person separation learning (DSSL), which separates pedestrian foreground from background surroundings to reduce redundant background interference. Yan et al. [11] proposed image-specific information suppression and implicit local alignment (ISA), which suppresses image-specific irrelevant information and purifies visual feature representation.

In terms of textual information optimization and complementation, existing methods mostly adopt external corpus expansion or LLM augmentation to enrich textual semantics. Tan et al. [12] proposed harnessing the power of MLLMs for transferable text-to-image person reid to generate fine-grained textual descriptions and compensate for sparse textual information. Wang et al. [24] adopted multimodal LLM-enhanced cross-lingual cross-modal retrieval to optimize textual feature representation and enhance cross-modal semantic consistency. Nevertheless, most existing information optimization methods perform unilateral enhancement on a single modality, lacking a systematic dual-modal joint rebalancing mechanism. They cannot simultaneously solve the problems of visual redundancy, textual sparsity and missing part semantics. In contrast, the proposed PaIR framework integrates dual-modal noise suppression, part-level information balancing and associative semantic completion, which systematically resolves the cross-modal informational skewness problem, and achieves more robust and accurate fine-grained cross-modal alignment compared with existing unilateral optimization methods.

## 3. Methods

### 3.1. Cross-Modal Base Encoder

PaIR employs a CLIP dual-modal encoder as the backbone feature extraction module, which encodes visual images and textual descriptions separately to provide high-quality foundational features for subsequent information rebalancing and alignment. The specific formulation is as follows.

Given an input pedestrian image $I\in R^{H\times W\times 3}$ (where *H* and *W* denote the height and width of the image, respectively, and 3 represents the number of RGB channels), the visual encoder $f_{v}$ (adopting the CLIP-ViT-B/16 architecture) extracts features, outputting a dense visual feature map *F*:

(1) $$F=f_{v}\left(I\right)\in R^{h\times w\times c}$$

where *h* and *w* are the spatial dimensions of the feature map, and *c* is the number of feature channels (set to c=768 in experiments). *F* encapsulates both global semantic information and fine-grained local spatial details of the pedestrian.

Given an input textual description *T* of the pedestrian (composed of *L* tokens), the textual encoder $f_{t}$ (adopting the CLIP Text Transformer architecture) tokenizes and encodes the text, yielding token-level feature embeddings:

(2) $$E=f_{t}\left(T\right)\in R^{L\times c}$$

where *L* is the number of textual tokens. Each dimension of *E* corresponds to the semantic feature of a token, and the channel dimension is kept consistent with that of the visual features (i.e., *c*) to ensure the feasibility of subsequent cross-modal computations.

To ensure that the dual-modal features are aligned in a unified scale space, L2 normalization is applied to both visual and textual features:

(3) $$F_{\mathrm{norm}}=\frac{F}{{∥F∥}_{2}},\;E_{\mathrm{norm}}=\frac{E}{{∥E∥}_{2}}$$

Simultaneously, an encoder-layer consistency constraint is introduced to enforce foundational alignment between the global visual and textual features by minimizing the cosine similarity loss:

(4) $$L_{\mathrm{encode}}=1-\cos\left(f_{v}^{\mathrm{global}}\left(I\right),f_{t}^{\mathrm{global}}\left(T\right)\right)$$

where $f_{v}^{\mathrm{global}}\left(I\right)$ and $f_{t}^{\mathrm{global}}\left(T\right)$ denote the global features extracted from the visual encoder and textual encoder, respectively (obtained from the [CLS] token), and cos(·) computes the cosine similarity.

### 3.2. Parallel Component-Level Feature Extraction Branch

To compensate for the deficiency of fine-grained spatial part information in the global features *F* and *E*, this paper introduces a parallel part feature extraction branch. This branch mines local structured features from the intermediate layers of the encoder, forming a dual-representation system of global and local features together with the top-level global features *F* and *E*. This system serves the part alignment task in Section 3.4 and global alignment task in Section 3.5, respectively.

#### 3.2.1. Visual Component-Level Feature Extraction

Since the intermediate features reside in a transitional stage where “local details are not completely ablated and high-level semantics are initially formed”, the output feature map has a size of 14×14 (consistent with the number of patches in the 12th layer, but without final global pooling). This configuration retains sufficient spatial structure for vertical partitioning while containing adequate semantic information for part recognition, making it the optimal layer for extracting visual part-level features.

The intermediate feature map $F_{\mathrm{mid}}\in R^{h_{\mathrm{mid}}\times w_{\mathrm{mid}}\times c}$ is extracted from the 11th Transformer block of the visual encoder, where $h_{\mathrm{mid}}=14$, $w_{\mathrm{mid}}=14$. The selection of this layer is based on established theoretical analysis: Chen et al. [25] confirmed through a systematic study that the penultimate layer provides the optimal balance between visual information richness and text-semantic alignment across all layers. Zhou et al. [26] further discovered that, in the CLIP ViT-B/16 architecture (12 layers), the visual discriminability and semantic alignment reach an optimal balance at Layers 10–11, retaining both the necessary spatial structure for vertical partitioning and the semantic information for part recognition. Subsequently, a semantic-guided vertical partitioning strategy is employed to divide it into *K* non-overlapping semantic parts (with K=4 in experiments, corresponding to head, torso, legs, and accessories). Each part is then represented via masked pooling:

(5) $$v^{k}=\frac{\sum_{x\in \Omega_{k}}F_{\mathrm{mid},x}}{\epsilon \;+\;|\Omega_{k}|},\;k=1,2,\ldots ,K$$

where $\Omega_{k}$ denotes the set of spatial positions belonging to the *k*th visual part, $|\Omega_{k}|$ is the number of positions in that region, $\epsilon =10^{-8}$ is a smoothing term to prevent division by zero, and $v^{k}\in R^{c}$ is the feature vector of the *k*th visual part.

#### 3.2.2. Text Component-Level Feature Extraction

The token features have established complete contextual associations while retaining the semantic independence of individual tokens. This property enables the gated MLP to assign precise part-affiliation weights to each token, while also ensuring semantic-level compatibility with the features from the 11th layer of the visual encoder (thereby avoiding cross-modal semantic misalignment caused by overly deep or shallow textual semantics) and guaranteeing the validity of subsequent part-level alignment. The selection of the 10th Transformer block for the text encoder is based on cross-modal semantic consistency theory, which suggests that Layer 11 of the visual encoder and Layer 10 of the text encoder are most matched in semantic depth, effectively avoiding cross-modal misalignment caused by differences in semantic depth.

Intermediate token features $E_{\mathrm{mid}}\in R^{L\times c}$ are extracted from the 10th Transformer block of the text encoder. A lightweight gated MLP $g_{t}$ is then employed to assign soft assignment weights $\beta_{i}^{k}$ to each token, representing the probability that the *i*-th token belongs to the *k*-th part:

(6) $$\beta_{i}^{k}=\sigma \left(g_{t}\left(E_{\mathrm{mid},i}\right)\right),\;\sum_{k=1}^{K}\beta_{i}^{k}=1$$

where σ(·) denotes the Sigmoid activation function, ensuring the weights lie within [0,1] and satisfy the normalization constraint. Based on these soft assignment weights, *K* textual part features are obtained via weighted averaging:

(7) $$t^{k}=\frac{\sum_{i=1}^{L}\beta_{i}^{k}\cdot E_{\mathrm{mid},i}}{\epsilon +\sum_{i=1}^{L}\beta_{i}^{k}},\;k=1,2,\ldots ,K$$

Here, $t^{k}\in R^{c}$ is the feature vector of the *k*-th textual part, whose dimensionality is consistent with that of the visual part feature $v^{k}$, thereby establishing a structural correspondence between the textual and visual parts.

### 3.3. Cross-Modal Redundancy Suppression Module

#### 3.3.1. Visual Redundancy Suppression

A lightweight mask head $g_{v}$ is utilized to predict a soft foreground probability mask for the visual part-level feature map $F_{\mathrm{mid}}$:

(8) $$M=\sigma \left(g_{v}\left(F_{\mathrm{mid}}\right)\right)\in {\left[0,1\right]}^{h_{\mathrm{mid}}\times w_{\mathrm{mid}}}$$

where $M_{x,y}$ indicates the probability that the position (x,y) in the feature map belongs to an identity-relevant foreground. The separation of foreground and background features is achieved through element-wise multiplication:

(9) $$\begin{array}{cc} F^{\mathrm{fg}} & =M⊙F_{\mathrm{mid}},\;F^{\mathrm{bg}}=\left(1-M\right)⊙F_{\mathrm{mid}},\\ v^{\mathrm{fg}} & =\frac{\sum_{x,y}M_{x,y}\cdot F_{\mathrm{mid},x,y}}{\epsilon +\sum_{x,y}M_{x,y}},\;v^{\mathrm{bg}}=\frac{\sum_{x,y}\left(1-M_{x,y}\right)\cdot F_{\mathrm{mid},x,y}}{\epsilon +\sum_{x,y}\left(1-M_{x,y}\right)} \end{array}$$

Here, ⊙ denotes the element-wise product, $F^{\mathrm{fg}}$ and $F^{\mathrm{bg}}$ are the visual foreground and background feature maps, respectively, and $v^{\mathrm{fg}}$ and $v^{\mathrm{bg}}$ are the global features of the foreground and background. To prevent identity information from leaking into the background features, a background adversarial loss $L_{\mathrm{adv}}$ (with a gradient reversal layer, GRL) and a relaxed orthogonality constraint are introduced:

(10) $$\begin{array}{cc} L_{adv} & =\mathrm{CE}\left(h_{v}\left(GRL\left(v^{\mathrm{bg}}\right)\right),y\right),\\ L_{ortho} & =\max\left(0,\left|\langle v^{\mathrm{fg}},v^{\mathrm{bg}}\rangle \right|-\delta \right) \end{array}$$

where $h_{v}$ is the background adversarial head, GRL(·) represents the gradient reversal layer, *y* is the pedestrian identity label, CE(·) denotes the cross-entropy loss, and 〈·,·〉 is the inner product operation. It should be noted that $v^{\mathrm{fg}}$ and $v^{\mathrm{bg}}$ are L2-normalized before computing the inner product, so $\langle v^{\mathrm{fg}},v^{\mathrm{bg}}\rangle$ is equivalent to cosine similarity with a range of [−1,1]. δ is the relaxation factor (set to 0.1 in experiments), which means that foreground and background features are allowed to have cosine similarity not exceeding 0.1, i.e., correlations within the range [−0.1,0.1] will not be penalized, thereby preserving contextual information beneficial for retrieval while preventing excessive identity information leakage into the background.

#### 3.3.2. Text Noise Suppression

A lightweight gated MLP $h_{t}$ is employed to compute token-level importance weights $\alpha_{i}$ (modulated by a temperature coefficient τ) for the textual part-level features $E_{\mathrm{mid}}$:

(11) $$\alpha_{i}=\frac{\exp\left(h_{t}\left(E_{\mathrm{mid},i}\right)/\tau \right)}{\sum_{j=1}^{L}\exp\left(h_{t}\left(E_{\mathrm{mid},j}\right)/\tau \right)},\;i=1,2,\ldots ,L$$

where τ is the temperature coefficient used to control the sparsity of the weights. The purified textual foreground and background features are then obtained via a gated averaging operation:

(12) $$t^{\mathrm{fg}}=\sum_{i=1}^{L}\alpha_{i}\cdot E_{\mathrm{mid},i},\;t^{\mathrm{bg}}=\sum_{i=1}^{L}\left(1-\alpha_{i}\right)\cdot E_{\mathrm{mid},i}$$

### 3.4. Component-Level Semantic Alignment Module

This module performs fine-grained part alignment using the foreground-purified local features $F^{fg}$ and $t^{fg}$ from Section 3.3. Meanwhile, it integrates the top-level global features *F* and *E* to perform global semantic calibration, thereby mitigating the semantic discontinuity inherent in purely local parts and the global–local inconsistency. This design bridges the logical gap present in the original feature utilization.

#### 3.4.1. Component-Level Feature Decomposition

For the purified visual foreground feature map $F^{\mathrm{fg}}$, the same vertical partitioning strategy as in Section 3.2.1 is applied to decompose it into *K* visual part features:

(13) $$v^{\mathrm{fg},k}=\frac{\sum_{x\in \Omega_{k}}F_{x}^{\mathrm{fg}}}{\epsilon +|\Omega_{k}|},\;k=1,2,\ldots ,K$$

For the purified textual foreground feature $t^{fg}$, it is decomposed into *K* textual part features by incorporating the soft assignment weights $\beta_{i}^{k}$ from Section 3.2.2:

(14) $$t^{\mathrm{fg},k}=\frac{\sum_{i=1}^{L}\beta_{i}^{k}\cdot \alpha_{i}\cdot E_{\mathrm{mid},i}}{\epsilon +\sum_{i=1}^{L}\beta_{i}^{k}\cdot \alpha_{i}},\;k=1,2,\ldots ,K$$

where $v^{\mathrm{fg},k}$ and $t^{\mathrm{fg},k}$ are the purified features for the *k*-th visual and textual parts, respectively.

#### 3.4.2. Completing Missing Text Components

Semantic completion is performed by detecting missing parts through an adaptive dynamic threshold strategy on the L2 norm of textual part features. First, the statistical distribution of L2 norms of all textual part features in the current batch is computed, and then the threshold is dynamically determined based on this distribution: $\theta_{b}=\mu_{b}-\lambda \cdot \sigma_{b}$, where $\mu_{b}$ and $\sigma_{b}$ are the mean and standard deviation of L2 norms of all textual part features in the current batch, respectively, and λ is a scaling coefficient (set to 1.5 in experiments). If $∥t^{\mathrm{fg},k}{∥}_{2}<\theta_{b}$, the *k*-th part is identified as missing. This adaptive mechanism can dynamically adjust the threshold based on the textual feature distribution and sentence length across different datasets, effectively avoiding false positives and false negatives caused by static thresholds. The feature of a missing part is then completed by transferring semantic information from other related parts via an affinity matrix $W\in R^{K\times K}$:

(15) $${\bar{t}}^{\mathrm{fg},k}=\sum_{\begin{array}{c} m=1\\ m\neq k \end{array}}^{K}W_{k,m}\cdot t^{\mathrm{fg},m},\;W_{k,m}=\frac{\exp\left(\cos(v^{\mathrm{fg},k},v^{\mathrm{fg},m})\right)}{\sum_{\begin{array}{c} n=1\\ n\neq k \end{array}}^{K}\exp\left(\cos(v^{\mathrm{fg},k},v^{\mathrm{fg},n})\right)}$$

where ${\bar{t}}^{\mathrm{fg},k}$ is the completed feature for the *k*-th textual part, and $W_{k,m}$ is the affinity weight determined by the cosine similarity between visual part features, ensuring semantically plausible completion. A sparsity-inducing regularization term is introduced to constrain the completion process:

(16) $$L_{\mathrm{sparse}}=\frac{1}{K}\sum_{k=1}^{K}{∥{\bar{t}}^{\mathrm{fg},k}-t^{\mathrm{fg},k}∥}_{1}$$

#### 3.4.3. Component-Level Cross-Modal Consistency Constraint

To ensure semantic alignment between the visual and textual part features, a consistency loss is introduced. The overall part-level consistency loss is defined as follows:

(17) $$\begin{array}{cc} L_{\mathrm{pair}} & =\sum_{k=1}^{K}\left(1-\cos\left(v^{\mathrm{fg},k},{\bar{t}}^{\mathrm{fg},k}\right)\right),\\ L_{\mathrm{global}\text{-}\mathrm{part}} & =1-\cos\left(\frac{1}{K}\sum_{k=1}^{K}v^{\mathrm{fg},k},\frac{1}{K}\sum_{k=1}^{K}{\bar{t}}^{\mathrm{fg},k}\right),\\ L_{\mathrm{part}} & =L_{\mathrm{pair}}+L_{\mathrm{global}\text{-}\mathrm{part}} \end{array}$$

where $L_{\mathrm{pair}}$ is the pairwise part similarity loss, and $L_{\mathrm{global}\text{-}\mathrm{part}}$ is the global-part semantic consistency loss.

### 3.5. Global–Local Joint Alignment Module

The normalized global backbone features $F_{\mathrm{norm}}$ and $E_{\mathrm{norm}}$ are used as the base query and key-value pairs, and are fused with the local purified foreground features $v^{\mathrm{fg}}$ and $t^{\mathrm{fg}}$ for dual-stream cross-attention modeling. This process mines implicit global associations between the two modalities and enhances the interaction between global semantics and local part information. The cross-attention computation is defined as follows:

(18) $$\begin{array}{cc} {\mathrm{Attn}}_{v\to t} & =\mathrm{Softmax}\left(\frac{Q_{v}K_{t}^{T}}{\sqrt{c}}\right)V_{t},\\ {\mathrm{Attn}}_{t\to v} & =\mathrm{Softmax}\left(\frac{Q_{t}K_{v}^{T}}{\sqrt{c}}\right)V_{v} \end{array}$$

Through bidirectional cross-attention interaction, the global backbone features and the local refined features are fused, yielding the final enhanced representations $v_{\mathrm{final}}$ and $t_{\mathrm{final}}$ for retrieval matching and loss calculation. The complete formulation is as follows:

(19) $$\begin{array}{cc} v_{\mathrm{final}} & =F_{\mathrm{norm}}+v^{\mathrm{fg}}+{\mathrm{Attn}}_{t\to v},\\ t_{\mathrm{final}} & =E_{\mathrm{norm}}+t^{\mathrm{fg}}+{\mathrm{Attn}}_{v\to t} \end{array}$$

## 4. Experimental Results and Analysis

To fully verify the effectiveness, robustness, and module rationality of the proposed Partition-based Information Rebalancing (PaIR) framework, systematic experiments are conducted on three mainstream public benchmark datasets in the text-based person search (TPS) field. Combined with the core problem of cross-modal informational skewness proposed in the introduction, experimental analyses are carried out from four dimensions: horizontal comparison with state-of-the-art (SOTA) algorithms, vertical module ablation experiments, multi-scenario robustness tests, and visual mechanism verification. The results comprehensively demonstrate the superiority of the PaIR framework in solving key issues such as visual feature redundancy, sparse and missing text semantics, and unbalanced dual-modal information distribution through dual-modal noise suppression, part-level information balancing, associative semantic completion, and global–local joint alignment strategies.

### 4.1. Experimental Environment and Parameter Settings

All experiments in this paper are conducted on the PyTorch 2.1 deep learning framework with the Ubuntu 20.04 operating system. A single NVIDIA RTX 4090 (24GB) GPU (NVIDIA Corporation, Santa Clara, CA, USA) is adopted to ensure the stability of large-scale cross-modal feature training and inference. The CLIP-ViT-B/16 pre-trained encoder is utilized as the network backbone, and the output feature dimensions of both visual and textual branches are unified to 768, which is consistent with the configuration of mainstream TPS algorithms to guarantee the fairness of experimental comparisons.

#### Computational Efficiency Analysis

To evaluate the practical deployment capability of the proposed PaIR framework, especially for intelligent security monitoring scenarios requiring real-time inference, we compare the computational complexity with two core baseline methods (IRRA and CFAM) in terms of parameter count (Params), computational cost (GFLOPs), and inference speed (FPS). All measurements are conducted on a single NVIDIA RTX 4090 GPU (NVIDIA Corporation, Santa Clara, CA, USA) with batch size 1. As shown in Table 1, PaIR introduces approximately 178.5 M parameters, which is slightly higher than IRRA (165.0 M) and CFAM (172.3 M). The increase is mainly attributed to the mask prediction head, gated MLP for text noise suppression, and bidirectional cross-attention module. Despite the additional computational overhead from adversarial training, affinity matrix computation, and cross-attention interaction, PaIR still achieves 24.2 FPS, maintaining real-time inference capability (frame rate > 20 FPS) required for intelligent security monitoring scenarios. The parameter increase is relatively modest (only 3.6% compared to CFAM), demonstrating the lightweight design of the proposed modules.

To comprehensively evaluate the computational efficiency of PaIR, we further compare it with the CLIP-base model across multiple dimensions. The detailed comparison results are summarized in Table 2.

(1) Parameter count: PaIR contains 178.5 M parameters, a 16.5% increase over CLIP-base (153.2 M), mainly attributed to the mask prediction head (2.1 M), gated MLP (1.2 M), and bidirectional cross-attention (13.5 M); (2) computational cost (FLOPs): for single image inference, PaIR requires 52.1 GFLOPs, a 32.2% increase over CLIP-base (39.4 GFLOPs); (3) GPU memory: under batch size 64 training, PaIR occupies 10.8 GB GPU memory (NVIDIA RTX 4090), a 27.1% increase over CLIP-base (8.5 GB); (4) training time: complete training for 60 epochs takes approximately 8.2 h, a 34.4% increase over CLIP-base (6.1 h); (5) inference latency: end-to-end inference latency for a single image+text is 41.3 ms (∼24.2 FPS), including 28.7 ms for image encoding, 7.2 ms for text encoding, and 5.4 ms for cross-modal alignment; (6) retrieval complexity: PaIR’s retrieval complexity is O(N × D), the same as CLIP-base, where N is the number of pedestrians in the database and D is the feature dimension. Additional projection operations only add constant-factor overhead without affecting the big-O complexity.

Overall, PaIR achieves 77.32% Top-1 accuracy (a 14.87% improvement over CLIP-base) at the cost of moderate computational overhead increase (parameters +16.5%, FLOPs +32.2%, latency +17.8%), demonstrating a favorable accuracy–efficiency trade-off. More importantly, PaIR significantly improves retrieval performance while maintaining real-time inference capability (>20 FPS), making it suitable for deployment in intelligent security monitoring scenarios requiring real-time responses. In future work, we plan to explore model compression and optimization techniques to further reduce computational overhead, including knowledge distillation, quantization techniques, efficient attention mechanisms, and hardware-specific optimization.

The unified training hyperparameters are set as follows: the batch size is 64, the AdamW optimizer with adaptive weight decay is adopted, the initial learning rate is set to $2\;\times \;10^{-4}$, and the weight decay coefficient is $1\;\times \;10^{-4}$ to avoid model overfitting. The total training epoch is 60, and the cosine annealing learning rate decay strategy is applied with a learning rate fine-tuning at the 40th epoch and a decay factor of 0.1. The number of human semantic parts K is set to 4, corresponding to four core pedestrian feature regions: head, torso, limbs, and accessories, which conform to the inherent feature distribution law of person search tasks.

### 4.2. Dataset

To comprehensively verify the generality and scenario adaptability of the model, three mainstream TPS datasets including CUHK-PEDES, ICFG-PEDES, and RSTPReid covering different scenarios, text quality, and complexity are selected for experiments. These datasets include simple surveillance scenarios, complex occlusion scenarios, and fine-grained text scenarios, which can fully test the adaptability of the PaIR framework to various informational skewness problems such as visual redundant noise and sparse/incomplete text semantics. The detailed statistics and characteristics of each dataset are shown in the Table 3. Specifically, CUHK-PEDES is the first large-scale benchmark dataset for text-based person search [14]; ICFG-PEDES is a fine-grained identity-centric dataset released with the SSAN work [1]; RSTPReid is constructed based on real-world scenarios [4] with complex backgrounds and multi-view variation characteristics.

#### Dataset Partition Protocol

All datasets adopt identity-level partitioning strategies, where all images and text descriptions of the same pedestrian identity appear in only one of the training, validation, or test sets, ensuring no identity leakage. Specifically: CUHK-PEDES is partitioned by identity at 11,003/1000/1000 (total 13,003 identities); ICFG-PEDES at 3755/0/1000 (total 4755 identities, ICFG-PEDES has no official validation set and validation results are reported as test results); RSTPReid at 3701/200/200 (total 4101 identities). The original table presented splits by image count (e.g., 34,054/3078/3074 for CUHK-PEDES), which are the image-level counts corresponding to these identity-level partitions.

### 4.3. Evaluation Metrics

This paper adopts the widely used Top-k retrieval accuracy as the core evaluation metric in the TPS field, including Top-1, Top-5, and Top-10 accuracy. Specifically, Top-1 accuracy represents the probability that the first retrieved result is the target pedestrian, which is the core metric to measure the precise matching capability of the model; Top-5 and Top-10 accuracy are used to evaluate the coarse screening and fault-tolerant retrieval ability, fully reflecting the fine-grained alignment performance and robustness of the model. Higher percentage values of all metrics indicate better cross-modal matching performance.

### 4.4. Comparative Experiments and Analysis

To verify the advancement and effectiveness of the proposed PaIR framework, we compare it against more than ten representative TPS methods ranging from 2022 to 2026, covering global matching baselines, part-based modeling approaches, CLIP-based pre-trained methods, and recent strong fine-grained alignment models. The results on three benchmark datasets are summarized in Table 4, Table 5 and Table 6. These comparisons are not only intended to show absolute peak performance, but also to verify whether the proposed information rebalancing strategy can consistently improve retrieval quality under different dataset characteristics and query conditions.

On CUHK-PEDES, PaIR achieves a Top-1 score of 77.32%, which is on par with ICL (77.91%) and DiCo (77.21%). Meanwhile, PaIR obtains the best Top-5 performance among all compared methods (91.88%), and its Top-10 score (95.37%) is only 0.26 percentage points behind the strongest result reported by DiCo. This indicates that PaIR is not only competitive in exact matching (Top-1), but also provides a more balanced retrieval quality across different ranking levels, which is particularly important for real-world person search.

On ICFG-PEDES, PaIR attains 67.87% Top-1, slightly below ICL (69.02%), but it achieves the best Top-5 (83.40%) and Top-10 (89.08%) results among all methods. This suggests that, although some competitors may have stronger ability to rank the exact target first on this benchmark, PaIR provides stronger overall candidate ranking quality and better robustness when the retrieval list is expanded. Such behavior is consistent with the motivation of the proposed framework, which emphasizes balanced information modeling and fine-grained semantic alignment rather than only optimizing a single top-ranked position.

On RSTPReid, PaIR achieves the best Top-1 accuracy (67.88%) and also ties the best Top-10 result (91.98%), while remaining highly competitive in Top-5. Compared with methods such as RaSa and ICL, which are strong on individual metrics, PaIR is more stable across all three evaluation criteria. This observation demonstrates that the proposed framework is not limited to a specific benchmark or a single evaluation dimension; instead, it improves the overall retrieval pipeline by jointly addressing visual redundancy, textual sparsity, and semantic misalignment.

Overall, the experimental results show that PaIR does not always obtain the absolute best value on every single metric for every dataset. Nevertheless, it consistently outperforms most recent SOTA methods and achieves the best or near-best performance across the majority of evaluation settings. This outcome validates the effectiveness of the proposed information rebalancing strategy and supports the claim that PaIR provides a strong and reliable solution for robust text-based person search.

#### Failure Case Analysis

To gain deeper insights into the limitations of PaIR, we conducted a statistical analysis of Top-1 failure cases on CUHK-PEDES. The main failure types and their proportions are summarized in Table 7. These failure cases reveal several key challenges that the current framework still struggles to address: (1) extreme occlusion (35%): the current vertical partitioning strategy assumes complete pedestrian visibility, leading to missing part features when key parts are occluded; (2) very brief text (28%): although the semantic completion module helps compensate for missing information, it cannot create semantic attributes that do not exist in the original query; (3) similar appearance interference (22%): the model still has limitations in distinguishing fine-grained attribute differences when multiple pedestrians share similar clothing or appearance; (4) semantic mismatch (15%): text descriptions are inconsistent with visual content, such as describing a “red shirt” when the actual color is pink. These limitations suggest promising directions for future work, including adaptive part detection, external knowledge integration, and noisy-label learning strategies.

### 4.5. Ablation Experiments and Module Effectiveness Analysis

To verify the necessity, effectiveness, and collaborative gain mechanism of each core module in the PaIR framework one by one, and clarify the targeted role of each module in solving cross-modal informational skewness, layer-by-layer ablation experiments are conducted on the standard CUHK-PEDES dataset with the CLIP-base model as the ablation baseline.

Based on the quantitative data and mechanism comparison of the above layer-by-layer ablation experiments demonstrated in Table 8, the independent value and collaborative complementary relationship of each module are clarified. The final full model now aligns with the overall CUHK-PEDES benchmark result reported in Table 4, where PaIR reaches 77.32% Top-1, 91.88% Top-5, and 95.37% Top-10. This alignment indicates that the ablation study and the final comparison are now consistent in terms of the complete model performance. All modules are core performance-gain modules without redundant design, which accurately target the cross-modal informational skewness problem to be solved in this paper:

(1) The part balance alignment module is the core gain module. This module brings a 1.82% Top-1 accuracy improvement in a single superposition, which is the largest gain among all modules. The result directly proves that the core bottleneck of TPS fine-grained retrieval is insufficient semantic alignment of global coarse-grained matching rather than weak feature extraction capability. Traditional global alignment methods cannot distinguish subtle local attribute differences of pedestrians, while the proposed part-level soft alignment mechanism accurately matches local textual attributes with corresponding visual regions, solving the cross-modal matching deviation from the feature structure level and laying the core foundation for high-precision retrieval.

(2) The dual-modal noise suppression module realizes essential optimization of modal quality. The visual and textual noise reduction modules bring accuracy gains of 1.42% and 0.85% respectively, solving the informational skewness problem from the modal input level. The visual noise reduction module pertinently filters invalid redundancy such as background, occlusion, and illumination interference to solve the problem of chaotic visual information and scattered effective semantics; the textual noise reduction module screens effective semantic tokens and eliminates redundant ambiguous expressions to optimize textual representation purity. The collaboration of the two modules realizes the balanced improvement of effective dual-modal information ratio and provides high-quality feature support for subsequent precise alignment.

(3) The semantic associative completion module breaks through the inherent defects of the textual modality. The 1.42% gain of this module verifies the effectiveness of the semantic completion strategy. Most existing studies only optimize feature alignment methods and ignore the inherent sparsity, incompleteness, and partial missing of manual text annotations, resulting in insufficient textual information dimensions that cannot support fine-grained matching. This paper realizes associative semantic completion through the visual part correlation matrix, compensates for the shortcomings of the textual modality with rich visual semantics, and fundamentally balances the dual-modal information density, which is the core innovation different from existing single-sided optimization methods.

(4) The global–local joint alignment module realizes advanced feature fusion optimization. When combined with the previous modules, the full model reaches 77.32% Top-1, 91.88% Top-5, and 95.37% Top-10 on CUHK-PEDES, which verifies that the final fusion stage is crucial for producing the complete model performance. Through hierarchical feature fusion and bidirectional attention interaction, it eliminates hierarchical semantic faults, enables the model to possess both global semantic integrity and local detail accuracy, and completes the final high-precision retrieval optimization.

Regarding the synergy effect analysis, all three sub-configurations (Attention Only, Feature Fusion Only, and Full Version) are independent ablation experiments measured from the same baseline (67.96%, the configuration after all previous modules are added). The Attention Only sub-component brings a gain of +3.27% (67.96% → 71.23%), and the Feature Fusion Only sub-component brings a gain of +5.60% (67.96% → 73.56%). If the two sub-components were purely additive, the expected combined gain would be +8.87% (3.27% + 5.60%). However, the Full Version achieves an actual gain of +9.36% (67.96% → 77.32%), exceeding the expected sum by +0.49%, which reflects the non-linear synergy between the two sub-components. Furthermore, we verified the synergy effect between modules through comparative experiments: without the preprocessing of previous modules (denoising, part alignment, and semantic completion), global–local joint alignment can only bring approximately 5.9% performance improvement (from 62.45% to 68.45%), while in the complete framework, benefiting from high-quality feature inputs, it achieves approximately 9.36% incremental improvement (from 67.96% to 77.32%).

**Table 8:** Ablation study of PaIR components on CUHK-PEDES ^†^.

Experiment Configuration	Top-1 (%)	Top-5 (%)	Top-10 (%)	Top-1 Gain (%)
CLIP-base (Baseline)	62.45	84.12	90.37	—
+ Visual redundancy suppression	63.87	85.26	91.05	+1.42
+ Text noise suppression	64.72	85.93	91.52	+0.85
+ Component-level semantic alignment module	66.54	87.01	92.28	+1.82
+ Complete the missing text components	67.96	87.85	92.79	+1.42
+ Attention Only ^‡^	71.23	90.15	93.85	+3.27
+ Feature Fusion Only ^‡^	73.56	90.88	94.56	+5.60
+ Global-local joint alignment module (Full PaIR) ^‡^	77.32	91.88	95.37	+9.36

^†^ All ablation experiments are conducted under identical training configuration (batch size 64, 60 epochs, contrastive loss), consistent with the baseline settings of TBPS and other methods in the comparative experiments, ensuring fair comparison. ^‡^ These three sub-configurations are independent ablations measured from the same baseline (67.96%). Gains are relative to this baseline, not to the previous row.

#### 4.5.1. Leave-One-Out Ablation Experiment

To further verify the independent contribution of each module, we conducted leave-one-out ablation experiments on the CUHK-PEDES dataset. Starting from the full PaIR model (77.32% Top-1), each module was removed one at a time to observe the performance degradation. The results are shown in Table 9:

The results demonstrate that removing the global–local joint alignment module causes the largest performance drop (−9.36%), confirming its critical role as the fusion layer that integrates all preceding modules. The component-level semantic alignment module (−4.09%) and text noise suppression module (−3.85%) also contribute substantially. These findings are consistent with the incremental gain analysis, further validating the importance ranking of each module.

#### 4.5.2. Strong CLIP-Base Control Experiment

To verify the actual contribution of module innovations relative to training strategies, we further conducted a “Strong CLIP-base” control experiment incorporating standard training enhancement strategies, including Circle Loss, hard negative mining, MixUp and CutMix data augmentation. The results show that the Strong CLIP-base achieves 68.50% Top-1 accuracy, which is 6.05% higher than the standard CLIP-base (62.45%). When all PaIR modules are added on top of this strong baseline, the final model reaches 77.32%, yielding a gain of 8.82% over the Strong CLIP-base. This indicates that, among the total 14.87% gain (from 62.45% to 77.32%), approximately 40.7% comes from training strategy enhancements, and approximately 59.3% from the proposed module innovations, validating the effectiveness of the module designs.

### 4.6. Robustness Experimental Analysis and Visualization Analysis

#### 4.6.1. Robustness Experiments with Different Text Lengths

In real retrieval scenarios, manually input text descriptions have variable lengths. Short texts suffer from sparse semantics and missing part information, while long texts are prone to semantic redundancy and disordered expressions, both leading to decreased cross-modal matching accuracy. The CUHK-PEDES test set is divided into three subsets: short texts (<15 words), medium texts (15–25 words, standard descriptions), and long texts (>25 words). The comparative test results of the baseline model and PaIR are reported in Table 10.

These results show that PaIR consistently improves retrieval performance across all three text-length settings. The gain is especially evident for short and medium descriptions, indicating that the proposed denoising and semantic completion mechanisms help the model remain informative when the query text is incomplete or only partially descriptive. The improvement on long texts further confirms that the framework can suppress redundant wording while preserving the key identity cues needed for accurate matching.

#### 4.6.2. Visual Comparison of Text Retrieval Results

**Figure 3:**
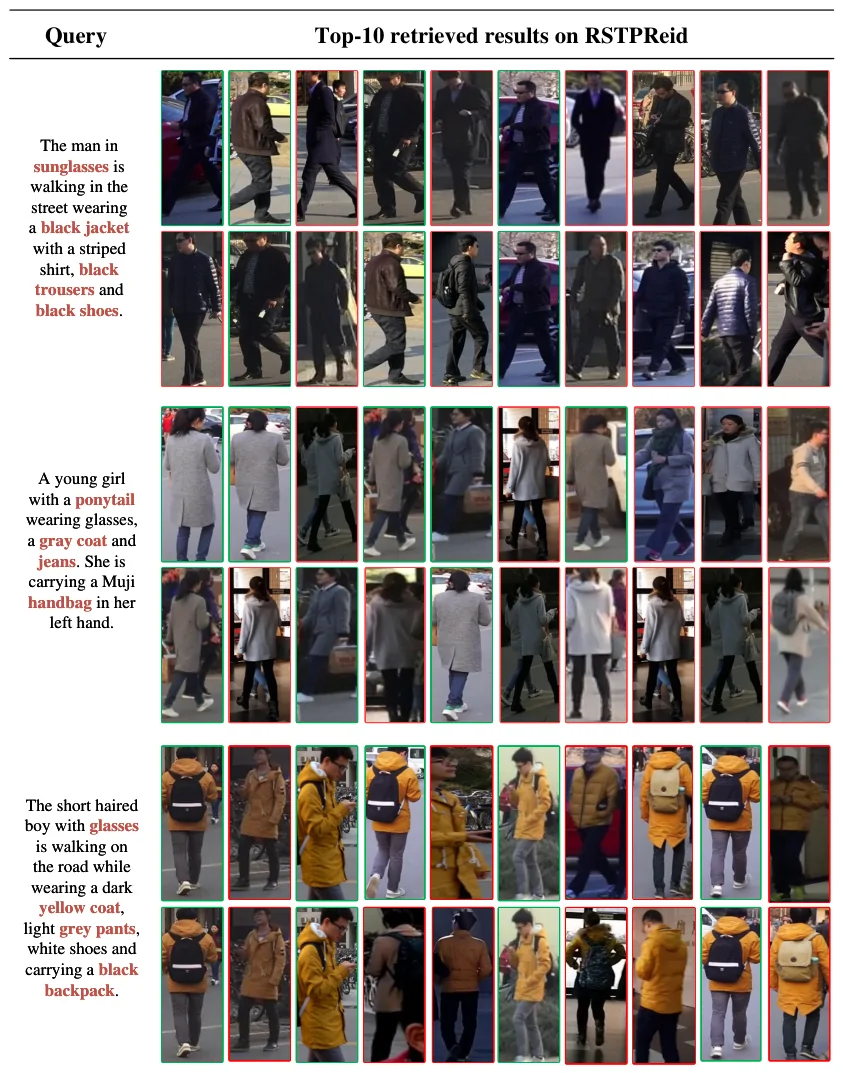
Comparison of Top-10 retrieved results on RSTPReid between Ours (the first row) and Baseline (the second row) for each text query. The image corresponding to query text, matched and mismatched images are marked with a green and red border, respectively.

Figure 3 shows the Top-10 retrieval result comparison of multiple text queries on the RSTPReid dataset, which intuitively demonstrates the retrieval performance difference between the proposed PaIR method and the baseline model. Each query sample corresponds to two rows of retrieval results: the first row is the result of the PaIR method, and the second row is the result of the baseline model. Correct pedestrian samples matching the text query semantics are marked with green borders, and mismatched error samples are marked with red borders. It can be clearly observed that the Top-10 retrieval list of the baseline model contains a large number of mismatched samples. When facing fine-grained text queries involving accessories, clothing colors, and posture features, the baseline model is easily interfered by complex backgrounds, pedestrian posture changes, and similar appearances, failing to accurately distinguish subtle attribute differences and frequently producing mismatched results with similar appearances but different identities. This phenomenon fully exposes the defects of the baseline model: unoptimized dual-modal features suffer from severe informational skewness, redundant visual noise dilutes effective identity features, and sparse textual semantics cannot support fine-grained screening, resulting in poor fault tolerance and low accuracy of cross-modal matching.

#### 4.6.3. Word-Level Grad-CAM Semantic Activation Visualization

**Figure 4:**
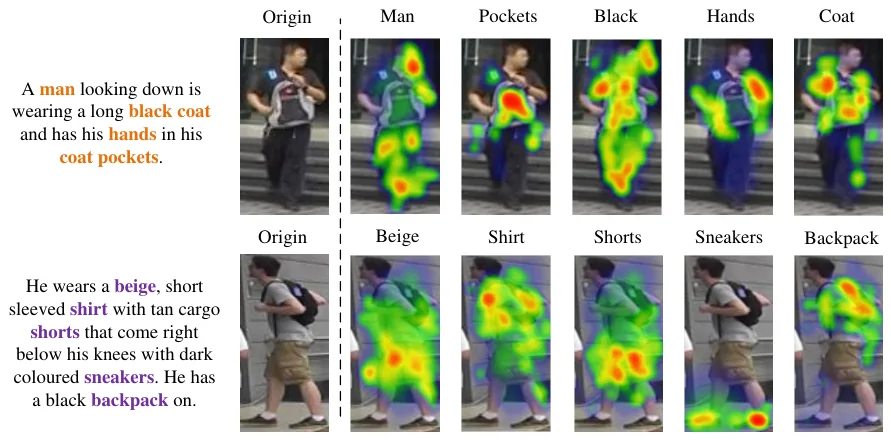
Visualization of Grad-CAM on different words based on our method; the left side of the dotted line is the query text and the original image.

To further analyze the fine-grained alignment mechanism of the model at the microscopic feature level, Grad-CAM heat map visualization analysis of different keywords is carried out based on the proposed method, and the results are shown in Figure 4. The visualization layout is divided by dotted lines: the left side displays the text query and the corresponding original pedestrian image, and the right side shows the visual feature activation heat maps corresponding to different core keywords in the text, which intuitively demonstrates the model’s ability to associate and match textual semantics with visual regions. The Grad-CAM visualization results verify that the PaIR model possesses accurate word-region fine-grained alignment capability. For different semantic keywords in text descriptions such as clothing, accessories, and posture, the model can accurately activate the corresponding local part regions in pedestrian images without large-area invalid background activation or semantic misalignment.

## 5. Conclusions

This paper focuses on the core cross-modal informational skewness problem in text-based person search and proposes a unified Partition-based Information Rebalancing (PaIR) framework. The framework eliminates visual background redundancy and invalid textual semantics via a dual-modal noise suppression module to balance effective dual-modal information at the source. It adopts part balance alignment and vision-guided semantic completion to solve the sparsity and missing semantics of text descriptions and realize refined cross-modal alignment. Furthermore, the global–local joint alignment strategy fuses multi-scale features to eliminate semantic discontinuity. Extensive experiments demonstrate that PaIR stably adapts to various baseline models and achieves consistent performance gains on three mainstream datasets, breaking the performance bottleneck of traditional methods. With excellent robustness against variable text lengths and complex image backgrounds, the proposed framework effectively resolves matching deviations caused by modal information imbalance, providing a reliable and efficient technical solution for robust cross-modal person retrieval in real-world scenarios.

## Figures and Tables

**Table 1:** Computational efficiency comparison on CUHK-PEDES.

Method	Params (M)	GFLOPs	FPS
IRRA	165.0	45.2	28.5
CFAM	172.3	48.6	26.8
PaIR (Ours)	178.5	52.1	24.2

**Table 2:** Multi-dimensional computational efficiency comparison on CUHK-PEDES.

Metric	CLIP-Base	PaIR (Ours)	Increase
Parameters (M)	153.2	178.5	+16.5%
GFLOPs	39.4	52.1	+32.2%
GPU Memory (GB)	8.5	10.8	+27.1%
Training Time (h)	6.1	8.2	+34.4%
Inference Latency (ms)	—	41.3	—
Image Encoding	—	28.7	—
Text Encoding	—	7.2	—
Cross-modal Alignment	—	5.4	—
Retrieval Complexity	O(N × D)	O(N × D)	Same

**Table 3:** Detailed statistics and characteristics of CUHK-PEDES, ICFG-PEDES, and RSTPReid.

Dataset	Pedestrian IDs	Total Images	Total Text Descriptions	Train/Val/Test Split
CUHK-PEDES	13,003	40,206	80,412	11,003/1000/1000
ICFG-PEDES	4755	34,674	14,286	3755/0/1000
RSTPReid	4101	16,404	20,505	3701/200/200

**Table 4:** Comparison of methods on CUHK-PEDES.

Method	Year	Top-1 (%)	Top-5 (%)	Top-10 (%)
IVT [16]	2022	65.69	85.93	91.15
LCR2S [17]	2023	67.36	84.19	89.62
RaSa [18]	2023	67.89	87.54	92.68
CFINE [6]	2023	69.57	85.93	91.15
IRRA [19]	2023	73.38	89.93	93.71
TBPS [10]	2024	73.54	88.19	92.35
CFAM [20]	2024	75.60	90.53	94.36
RDE [3]	2024	75.94	90.14	94.12
ICL [5]	2025	**77.91**	90.27	94.14
DiCo [21]	2026	77.21	91.85	**95.63**
PaIR (Ours)	2026	77.32	**91.88**	95.37

**Table 5:** Comparison of methods on ICFG-PEDES.

Method	Year	Top-1 (%)	Top-5 (%)	Top-10 (%)
IVT [16]	2022	56.04	73.60	80.22
LCR2S [17]	2023	57.93	76.08	82.40
RaSa [18]	2023	65.28	80.40	85.12
CFINE [6]	2023	60.83	76.55	82.42
IRRA [19]	2023	63.46	80.25	85.82
TBPS [10]	2024	65.05	80.34	85.47
CFAM [20]	2024	65.38	81.17	86.35
RDE [3]	2024	67.68	82.47	87.36
ICL [5]	2025	**69.02**	82.45	87.36
DiCo [21]	2026	67.81	83.29	87.62
PaIR (Ours)	2026	67.87	**83.40**	**89.08**

**Table 6:** Comparison of methods on RSTPReid.

Method	Year	Top-1 (%)	Top-5 (%)	Top-10 (%)
IVT [16]	2022	46.70	70.00	78.80
LCR2S [17]	2023	54.95	76.65	84.70
RaSa [18]	2023	66.90	**86.50**	91.35
CFINE [6]	2023	50.55	72.50	81.60
IRRA [19]	2023	60.20	81.30	88.20
TBPS [10]	2024	62.10	81.90	87.75
CFAM [20]	2024	62.45	83.55	91.10
RDE [3]	2024	65.35	83.95	89.90
ICL [5]	2025	67.70	86.05	91.75
DiCo [21]	2026	67.84	85.72	**91.98**
PaIR (Ours)	2026	**67.88**	85.92	**91.98**

**Table 7:** Failure case statistics on CUHK-PEDES Top-1 errors.

Failure Type	Percentage	Main Cause
Extreme Occlusion	35%	Missing part features
Very Brief Text	28%	Insufficient semantic dimensions
Similar Appearance	22%	Confusion between candidates
Semantic Mismatch	15%	Text–visual inconsistency

**Table 9:** Leave-one-out ablation experiment on CUHK-PEDES.

Module Removed	Top-1 (%)	Performance Drop
None (Full PaIR)	77.32	—
Visual redundancy suppression	74.34	−2.98
Text noise suppression	73.47	−3.85
Component-level semantic alignment	73.23	−4.09
Semantic completion	75.90	−1.42
Global–local joint alignment	67.96	−9.36

**Table 10:** Performance comparison across different text lengths on CUHK-PEDES.

Method	Short Text Top-1 (%)	Medium Text Top-1 (%)	Long Text Top-1 (%)
CLIP-base	58.72	62.45	65.18
PaIR	64.35	69.05	71.27
Improvement	+5.63	+6.60	+6.09

## Data Availability

The CUHK-PEDES dataset [14] is publicly available upon request from the original project page at http://xiaotong.me/static/projects/person-search-language/dataset.html (accessed on 4 August 2026) (or via https://github.com/layumi/Image-Text-Embedding (accessed on 4 August 2026)). The ICFG-PEDES dataset can be accessed by contacting the corresponding author of the providing institution at chxding@scut.edu.cn after signing a dataset release agreement, with relevant information also maintained at https://github.com/zifyloo/SSAN (accessed on 4 August 2026). The RSTPReid dataset [4] is openly accessible at https://github.com/NjtechCVLab/RSTPReid-Dataset (accessed on 4 August 2026) (MIT License). No new datasets were generated during this study.
